# Molecular Epidemiology and Evolutionary Analysis of Avian Influenza A(H5) Viruses Circulating in Egypt, 2019–2021

**DOI:** 10.3390/v14081758

**Published:** 2022-08-11

**Authors:** Naglaa M. Hagag, Nahed Yehia, Mohamed H. El-Husseiny, Amany Adel, Azhar G. Shalaby, Neveen Rabie, Mohamed Samy, Motaz Mohamed, Amal S. A. El-Oksh, Abdullah Selim, Abdel-Satar Arafa, Samah Eid, Momtaz A. Shahein, Mahmoud M. Naguib

**Affiliations:** 1 Reference Laboratory for Veterinary Quality Control on Poultry Production, Animal Health Research Institute, Agriculture Research Center, Giza 12618, Egypt; 2 Virology Department, Animal Health Research Institute, Agriculture Research Center, Giza 12618, Egypt; 3 Zoonosis Science Center, Department of Medical Biochemistry and Microbiology, Uppsala University, 75121 Uppsala, Sweden

**Keywords:** avian influenza, H5N8, virus evolution, genetic diversity, Egypt, poultry

## Abstract

The highly pathogenic avian influenza (HPAI) H5N8 virus was first detected in Egypt in late 2016. Since then, the virus has spread rapidly among different poultry sectors, becoming the dominant HPAI H5 subtype reported in Egypt. Different genotypes of the HPAI H5N8 virus were reported in Egypt; however, the geographic patterns and molecular evolution of the Egyptian HPAI H5N8 viruses are still unclear. Here, extensive epidemiological surveillance was conducted, including more than half a million samples collected from different poultry sectors (farms/backyards/live bird markets) from all governorates in Egypt during 2019–2021. In addition, genetic characterization and evolutionary analyses were performed using 47 selected positive H5N8 isolates obtained during the same period. The result of the conducted surveillance showed that HPAI H5N8 viruses of clade 2.3.4.4b continue to circulate in different locations in Egypt, with an obvious seasonal pattern, and no further detection of the HPAI H5N1 virus of clade 2.2.1.2 was observed in the poultry population during 2019–2021. In addition, phylogenetic and Bayesian analyses revealed that two major genotypes (G5 and G6) of HPAI H5N8 viruses were continually expanding among the poultry sectors in Egypt. Notably, molecular dating analysis suggested that the Egyptian HPAI H5N8 virus is the potential ancestral viruses of the European H5N8 viruses of 2020–2021. In summary, the data of this study highlight the current epidemiology, diversity, and evolution of HPAI H5N8 viruses in Egypt and call for continuous monitoring of the genetic features of the avian influenza viruses in Egypt.

## 1. Introduction

In the last decade, influenza A viruses of subtype (H5Nx) have continued to circulate in migratory birds and spread to infect domestic poultry in many countries worldwide [1,2]. The highly pathogenic avian influenza (HPAI) H5N8 virus was isolated for the first time in 2010 in China as a result of the genetic reassortment of different avian influenza subtypes [3]. In early 2014, a new reassortant of the HPAI H5N8 virus was reported in South Korea [4], which further spread via migratory birds to Europe and North America, causing multiple outbreaks that clustered phylogenetically as clade 2.3.4.4a [5,6,7]. In 2016/2017, novel reassortants of the HPAI H5N8 virus, with a different gene constellation known as clade 2.3.4.4b, were detected in migratory birds in several countries around the globe [8,9,10]. Since then, the virus has undergone continual evolutionary divergence via reassortment with other influenza A subtypes, resulting in the emergence of various genotypes and further spreading to domestic birds [8]. Genetically different reassortant combinations of six distinct genotypes of H5N8 were commonly detected [8]. In February 2021, the first human case of HPAI H5N8 virus infection was reported in Russia in two farmers with a history of contact with infected birds [11].

In Egypt, the HPAI H5N8 virus was originally detected in migratory bird (common coots—*Fulica atra*) in late 2016 [12]. The original virus was phylogenetically closely related to other H5N8 viruses of clade 2.3.4.4b detected in Russia in 2016 [13]. Targeted AIV surveillance was performed in response to the virus detection in late 2016–2017 [14,15]. Six genotypes of the HPAI H5N8 virus were reported in migratory and domestic birds in Egypt based of their whole genome sequence [13,14,15,16,17]. Thereafter, the virus spread in a very short time among domestic poultry populations in different governorates in Egypt, posing a great threat to the poultry industry [14,15]. The Egyptian HPAI H5N8 virus was also involved, via reassortment with the Egyptian low pathogenic avian influenza (LPAI) H9N2 virus, in the emergence of novel HPAI H5N2 viruses in Egypt in 2018/2019 [18,19]. The HPAI H5N8 viruses detected in Europe in 2020 were found to be phylogenetically related, based on the HA (haemagglutinin) gene sequence, to the HPAI H5N8 viruses isolated in Egypt in 2019 [7,20]. Epidemiologic data suggested that the HPAI H5N8 virus(clade 2.3.4.4b) has replaced the Egyptian H5N1 virus (clade 2.2.1.2), becoming the most commonly detected H5 subtype in Egyptian poultry sectors [21].

It is currently evident that HPAI H5N8 viruses circulate endemically in domestic poultry species in Egypt [22,23]. However, the epidemiology and genetic evolution of the Egyptian HPAI H5N8 viruses are not yet well understood. Hence, in order to further understand the diversity and evolution of HPAI H5N8 viruses circulating in Egyptian poultry, more than half a million samples, collected from poultry farms, backyards, and live bird markets (LBMs) in Egypt between 2019 and 2021 as a part of the surveillance program, were analyzed. In addition, extensive genetic analysis was performed to assess the phylogenetic and genetic structure of the Egyptian HPAI H5N8 viruses. Finally, the molecular dating was estimated and selection pressure was calculated using sequences generated in this study, along with all sequences publicity available for the Egyptian HPAI H5N8 viruses.

## 2. Materials and Methods

### 2.1. Samples Collection

A total of 506,097 swabs were collected from 30,159 commercial poultry (chickens, ducks, and turkeys) farms in Egypt—a total of 29,468 farms as part of active surveillance, (planned surveillance, and pre-slaughter check) and 691 farms as part of passive surveillance (due to reported cases with symptoms) as shown in Supplementary Table S1. Additionally, 8276 swabs from 783 backyards and 34,905 swabs from 984 live bird markets (LBMs) were retrieved. A total of 10 to 20 individual tracheal and/or cloacal swabs collected from each farm, backyard, or LBM were pooled together and treated as one sample. Samples were obtained from 27 governorates in Upper and Lower Egypt (Supplementary Table S1) and collected as a part of an active and passive avian influenza virus surveillance program in Egypt performed by the Reference Laboratory of Veterinary Quality Control on Poultry Production (RLQP), Animal Health Research Institute, Egypt, and the General Organization for Veterinary Services (GOVs). The epidemiological data of the collected samples and the H5N8 avian influenza positive samples are provided in Supplementary Tables S1 and S2.

### 2.2. Molecular Detection and Virus Isolation

Briefly, viral RNA from each sample was extracted using the QIAamp Viral RNA Mini Kit (Qiagen, Hilden, Germany) according to the manufacturer’s instructions. All extracted RNAs were initially examined for the matrix (M) gene of influenza A viruses using standard quantitative reverse transcription polymerase chain reaction (RT-qPCR) [24]. Positive influenza M RNAs were then tested using gene-specific RT-qPCR assays for the hemagglutinin (HA) and neuraminidase (NA) gene segments of the AIV H5, and N1, N2, and N8, respectively [25]. The RT-qPCR reactions were conducted using the Stratagene MX3005P real-time PCR machine (Agilent, Santa Clara, CA, USA). Further, samples that were found RT-qPCR positive for influenza genes were inoculated into the allantoic cavity of 9 to 11-day-old specific pathogen-free (SPF) embryonated chicken eggs (ECEs) according to the standard protocols of the OIE (World Organization for Animal Health) diagnostic manual [26]. Allantoic fluid from inoculated eggs was harvested 36–48 h post inoculation. Virus detection in the allantoic fluid was initially performed by hemagglutination assay with 1% chicken red blood cells and then verified by RT-qPCR for the AIV HA and NA genes.

### 2.3. Sequencing and Phylogenetic Analyses

Complete gene segments of the HA and NA were amplified for 47 and 19 positive isolates, respectively, using primers previously described by Hoper et al. [27]. In addition, whole-genome amplification of four representative H5N8 viruses was conducted using a set of forward and reverse primers for each gene segment, as previously described [27]. Briefly, all specific RT-PCR genomic segments were size-separated by agarose gel electrophoresis, excised, and purified using the QIAquick Gel Extraction Kit (Qiagen, Hilden, Germany). Further, purified PCR products were used directly for cycle sequencing reactions using the BigDye Terminator v3.1 Cycle Sequencing Kit (Applied Biosystems, Waltham, MA, USA). Sequence amplified products were cleaned up using a Centrisep spin column (Thermo Fisher, Waltham, MA, USA) and processed using an ABI PRISM 3100 Genetic Analyzer (Life Technologies, Carlsbad, CA, USA). Obtained sequences were assembled and edited using Geneious Prime 2021.1.1 (https://www.geneious.com). A Blast search was performed using the Basic Local Alignment Search Tool (BLASTn) at NCBI https://blast.ncbi.nlm.nih.gov/Blast.cgi (accessed on 1 March 2022), and sequences established in this study were submitted to GenBank under accession numbers shown in Supplementary Table S2.

Further, genetic sequences of representative global HPAI H5N8 viruses from clade 2.3.4.4a and 2.3.4.4b, as well as for Egyptian HPAI H5N8 viruses, were retrieved from GISAID platforms (GISAID, http://www.gisaid.org, accessed 1 May 2022). Representative viruses were selected based on geographical locations and availability of whole genome sequences to obtain a similar representation in all gene segment analyses. The nucleotide sequences of retrieved viruses and viruses obtained in the current study were aligned using MAFFT [28]. Phylogenetic trees, for all gene segments except the HA and NA, were constructed after the selection of the best-fitted model, by employing maximum likelihood methodology based on the Akaike criterion using IQ-tree software version 1.1.3 [29]. Finally, phylogenetic trees were annotated and visualized using FigTree v1.4.2 software (http://tree.bio.ed.ac.uk/software/figtree/, accessed on 5 April 2022) and Inkscape 1.0 (Available at https://inkscape.org).

### 2.4. Times of Most Recent Common Ancestor (tMRCAs)

The HA and NA sequences, including all Egyptian HPAI H5N8 viruses and representative sequences from other countries, were downloaded from the GISAID (GISAID, http://www.gisaid.org, accessed on 1 May 2022) and aligned with sequences generated in this study. Full or near full length sequences of HA (n = 1588) and NA (n = 1313), in addition to sequences generated in this study, were used for this analysis. Further, concatenated sequences including the coding sequences of the eight segments were generated using Geneious Prime 2022.1.1 (https://www.geneious.com), including viruses in this study, all whole genome available Egyptian HPAI H5N8 viruses, and representative global H5N8 viruses.

Molecular dating was performed for each of the HA, NA, and the concatenated tree using the Bayesian Evolutionary Analysis Sampling Trees BEAST version 1.8.4. After the selection of the best-fitting model, HA, NA, and the concatenated trees were performed using gamma distributed rates-across-sites, a strict molecular clock model, and the birth-death skyline serial model using simulations for 100 million generations with sampling every 10,000 steps. The log and trees files from two separate MCMC runs were merged using the LogCombiner (v1.8.4) program after removal of 10% of the chain as burn-in. All parameters were assessed using the Tracer (v1.7.2) program. Maximum clade credibility (MCC) trees were then visualized using the TreeAnnotator (v1.8.4) program. 

### 2.5. Selection Pressure

Selection pressure modes were applied on alignments of 242 and 146 sequences for HA and NA of the Egyptian H5N8 viruses, respectively, using the codon-based approach available at Datamonkey 2.0 [30]. Evidence of positive selection was determined at dN/dS > 1.0 and *p*-value < 0.05, selection of a site was accomplished using the pervasive evolutionary method represented in fixed-effects likelihood (FEL), and the episodic evolutionary method as the mixed effects model of evolution (MEME) method.

## 3. Results

### 3.1. Active and Passive Surveillance

**Figure 1:**
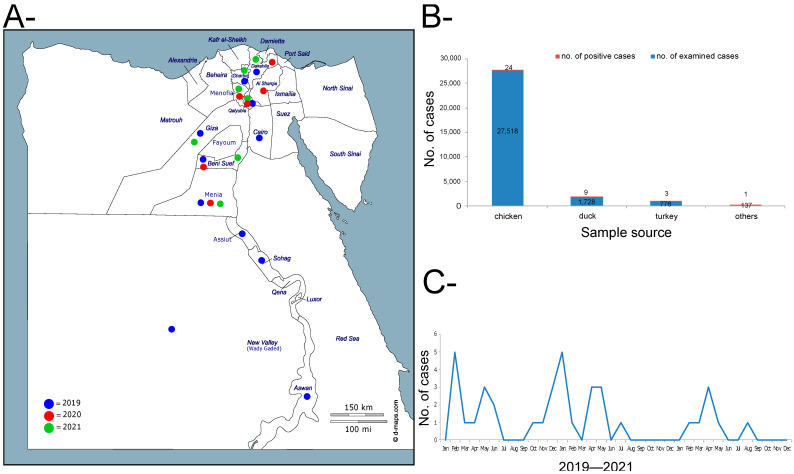
Surveillance and epidemiological features of H5N8 in Egypt commercial farms, 2019–2021. (**A**) Geographical distribution of HPAI H5N8 viruses in Egypt during 2019–2021. (**B**) Number of samples collected from each species indicating the total tested and the total detected as positive for the H5N8 virus. “Others” indicates other sources rather than chicken, duck, and turkey (e.g., environmental samples). (**C**) Distribution of positive H5N8 per month over 2019–2022.

In total, only 17 farms (0.06%) of apparently healthy birds were found to be positive for the influenza H5N8 subtype among the 29,468 examined farms as a part of the active surveillance program in Egypt in 2019–2021. In addition, 20 farms (2.89%) tested positive from the 691 examined farms as part of the passive surveillance. Positive farms were distributed over 13 governorates in Upper and Lower Egypt during 2019–2021, with geo-prevalence of 48.1% (Figure 1A,B). From the 37 positive farms, the majority of positive results were found in chicken (n = 24), then duck (n = 9), followed by turkey (n = 3), and one was un-identified, but found positive for the influenza H5N8 subtype. (Figure 1B). A clear seasonal variation was found in this three-year period, where a high number of positive cases were detected during the winter season—late November to April (Figure 1C). Moreover, 27 (3.44%) and 86 (9.75%) samples were found positive for HPAI H5N8 viruses in backyards and LBMs, respectively. Additionally, two HPAI H5N2 cases were recorded in two LBMs in Giza in October and November 2021 in mixed species, (chicken, duck, and turkey).

### 3.2. Genetic Diversity and Selection Pressure

Based on the genetic variation of the internal gene segments, six HPAI H5N8 genotypes were found in Egypt. Among them, genotypes G5 and G6 are the currently dominant genotypes circulating among the Egyptian poultry farms in 2019–2021. The hemagglutinin (HA) revealed the presence of multiple basic amino acid motif PLREKRRKR/GLF at the HA cleavage site, confirming a highly pathogenic status. The receptor binding pocket of the HA protein of all Egyptian isolates displayed amino acids H103, N182, G221, Q222, and G224 (H5 numbering), suggesting an avian-like α2,3-sialic acid receptor binding preference [31,32]. Genetically, G5 genotype viruses displayed R72S and N183S substitutional mutations in the HA coding segment, respectively. The Egyptian H5N8 G6 genotype is characterized by unique amino acids T140A and V522A (H5 numbering); in addition, Egyptian H5N8 viruses from 2021 are characterized by the N236D substitution mutation. No substitutional amino acid mutations related to oseltamivir or amantadine resistance were detected in the NA and MA, respectively (Table 1).

Further, positive selection pressure for hemagglutinin occupied six amino acid residues, including the amino acid residue 94 at antigenicity associated site A that showed a strong positive selective pressure by both of the analytical methods used for selection. Moreover, 24 residues on neuraminidase have possessed positive selection pressure, distributed along the NA molecule (Table 2).

### 3.3. Phylogenetic Characterization and Molecular Dating

**Figure 2:**
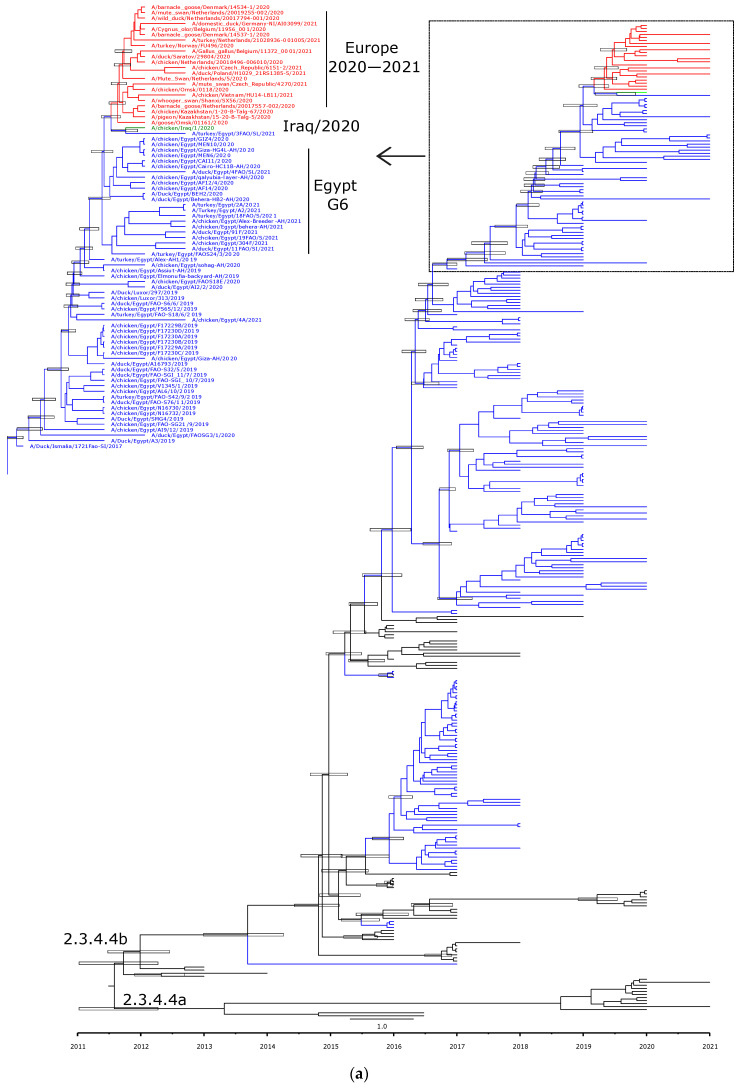

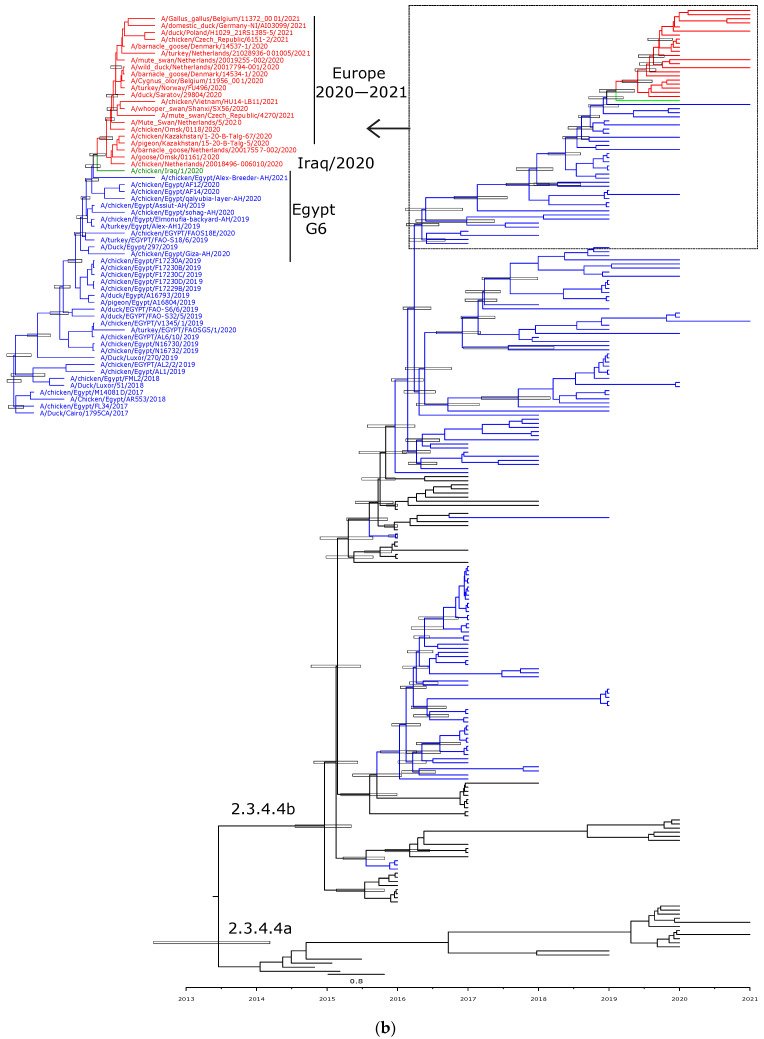
Time-scaled Bayesian maximum clade credibility tree of the (**a**) HA gene and (**b**) NA gene segment of HPAI H5N8 viruses from Egypt (blue) and representative H5N8 viruses from clade 2.3.4.4a and 2.3.4.4b. Node bars represent 95% Bayesian credible intervals for estimates of common ancestry, which are shown on the main nodes.

**Figure 3:**
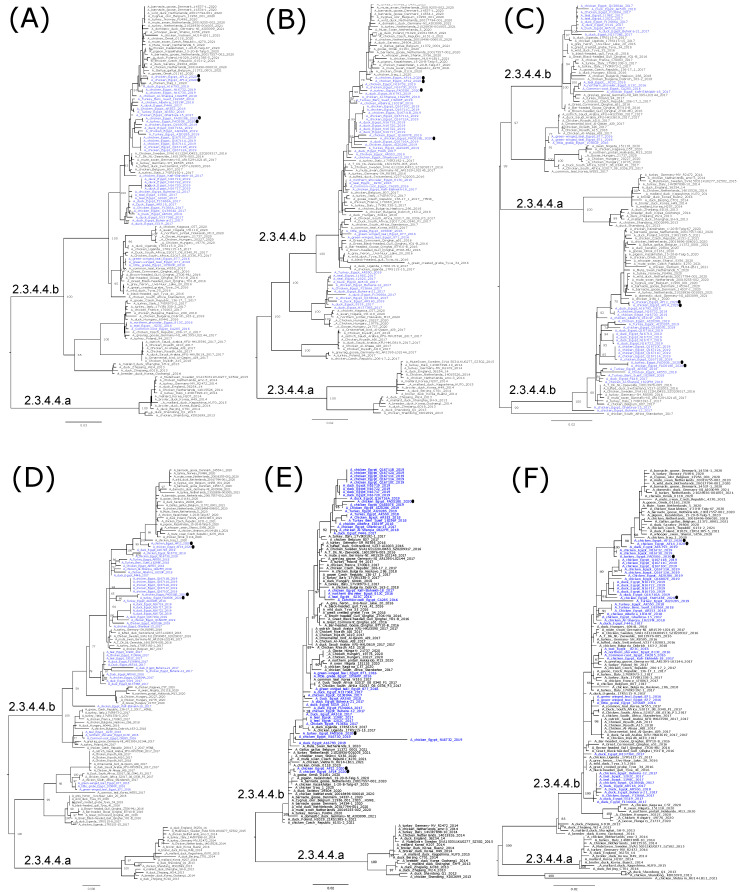
Phylogenetic relationships of the PB2 (**A**), PB1 (**B**), PA (**C**), NP (**D**), M (**E**), and NS (**F**) gene segments of Egyptian and representative H5N8 viruses. Egyptian H5N8 viruses are colored in blue, and viruses generated by the whole genome sequence in this study are shown by black dots. Trees were generated, after the selection of the best-fitted model, by employing maximum likelihood methodology based on the Akaike criterion using IQ-tree software version 1.1.3 [29].

**Figure 4:**
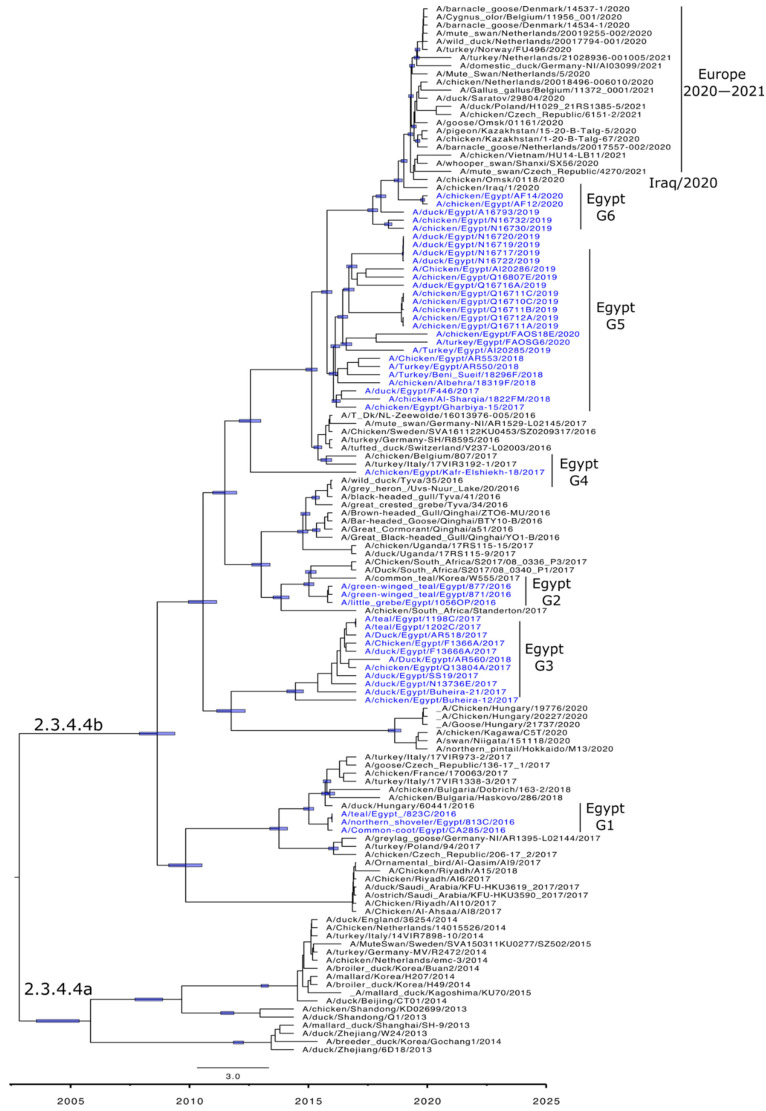
Time-scaled Bayesian maximum clade credibility tree of the concatenated whole genome of HPAI H5N8 viruses from Egypt (blue) and representative H5N8 viruses. Node bars represent 95% Bayesian credible intervals for estimates of common ancestry and are shown on the main nodes. Genotyping of the Egyptian viruses is based on sequence similarity and years of detection.

Phylogenetic analyses are in accord with the genetic characterization, indicating that the Egyptian HPAI H5N8 viruses are found in six different lineages, confirming the detection of at least six genotypes of HPAI H5N8 viruses in Egypt (Figure 2a,b, Figure 3A–F and Figure 4). In addition, the topology of the trees revealed a phylogenetic relatedness of the Egyptian to the recent European HPAI H5N8 viruses from 2020–2021 in all included gene segments (Figure 2a,b, Figure 3A–F and Figure 4). Moreover, the concatenated phylogenetic tree showed the same relatedness with the European viruses.

Molecular clock-based trees were generated based on the HA and NA gene segments, as well as concatenated sequencing of the whole gene segments. As we described previously [13], the estimation of time of the most recent common ancestor (tMRCAs) revealed that Egyptian viruses were introduced in late 2016, likely from Russia. This was also confirmed in the present study including HA, NA, and the concatenated whole genome. Additionally, the Egyptian HPAI H5N8 viruses from 2019 are shown to be the most likely ancestor of the recent HPAI H5N8 viruses spread in Europe over the last two years (Figure 2a,b and Figure 4).

## 4. Discussion

Influenza A (H5N8) viruses have been the dominant H5 subtype reported among different bird species around the globe since 2014 [8]. During 2020–2021, HPAI H5N8 viruses were frequently isolated from migratory birds and domestic poultry in several countries worldwide [7,33]. Recently, the HPAI H5N8 virus was detected for the first time in humans working at an infected chicken farm in Russia [11]. Since its introduction into Egypt in late 2016, HPAI H5N8 viruses have begun to replace the previously endemic HPAI H5N1 viruses in both commercial farms and backyard sectors [21]. The aim of the present study was to determine (i) the epidemiological picture, (ii) the genetic and phylogenetic features, and (iii) the evolution and selection pressure of HPAI H5N8 viruses isolated from Egypt in 2016–2021.

In the current study, no positive HPAI H5N1 viruses of clade 2.2.1.2 were detected in poultry sectors in Egypt during 2019–2021. This confirms our [21] and others’ studies [34] that showed no further HPAI H5N1 virus circulation in the poultry population in Egypt in the period between mid-2017 to mid-2020. Further, lower numbers (17/29,468; 0.06%)) of HPAI H5N8 positive farms were found in 22/27 governorates in Lower and Upper Egypt in the active surveillance compared to 20/691 (2.89%) farms as part of the passive surveillance. Usually, passive surveillance is based on clinically suspected cases, which justifies the higher incidence of positive H5 virus results. Further, higher incidence found in backyard and LBMs compared to poultry farms could be due to the vaccination programs implemented in farms against H5 subtypes, where typically, no vaccination occurs in backyards. Recently, a higher detection rate of 41.7% was reported by Salaheldin et al., 2022 [35] from only 211 tested poultry farms in Egypt. This highlights the need for collaboration between the different laboratories in Egypt to obtain an in-depth genetic feature of circulating H5 viruses.

The genetic and phylogenetic characterizations performed in this study revealed at least six genotypes of HPAI H5N8 viruses, as described previously [13] [14,16,34]. Here, out of six genotypes, only two genotypes (G5, G6) were found to still be circulating among poultry sectors in Egypt. Genetically, The HA of the Egyptian genotypes G5 and G6 displayed amino acid mutations R72S/N183S and T140A, respectively, in their antigenic sites, underlining the importance of continuous genetic monitoring and updating of the H5 vaccination program in Egypt. Positive selection pressure has been determined at amino acid site 94 of the HA, which is one of the characteristic antigenic sites of the H5 viruses [31,36], in addition to other sites that are adjacent to the antigenic and receptor-binding sites of the HA. Viruses are exposed to selection pressure during replication through the years, and this can be caused by different factors, the most common being the vaccination pressure that induced mutations on the surface antigens HA and NA [37]. In addition, accumulation of positive selection pressure sites have a crucial impact on the evolution of the virus; it may be a principle cause of releasing more phenotypes that may possess variable pathogenic and antigenic characteristics, in addition to the changes in the avidity of virus attachment and adaptation to the different hosts [38,39]. Further, molecular dating analysis indicates that the Egyptian HPAIV H5N8 viruses are the most likely ancestors of the recent HPAI H5N8 virus spread in Europe over the last two years 2020/2021. This might be due to the back-spread of H5 viruses from domestic poultry to migratory birds in Egypt, along with further dissemination to different countries.

In conclusion, the extinct of HPAI H5N1 viruses in Egypt and the dominance of two genotypes of HPAI H5N8 in the poultry sectors in Egypt calls for the re-evaluation of the vaccination policy in Egypt. Updating the vaccine seed in a timely manner is important to ensure the effectiveness of the vaccine in controlling HPAI H5 virus in Egypt. In countries applying vaccination against HPAIV H5 virus, such as China, the vaccine seeds had been updated many times and were shown to be protective [40]. Currently, vaccination against the HPAI virus in Egypt is based on several vaccination regimes, including H5 viruses from different clades (clade 2.2.1.2, clade 2.3.2.1c, and clade 2.3.4.4). Based on the current study, the vaccine seed should be updated to minimize the antigenic mismatch with the circulating strains. Vaccination against the HPAI virus in Egypt should be done in a consistent manner to overcome the vaccination failure due to unsuitable vaccination programs or the lack of vaccination in backyard poultry—and some broiler—farms. In addition, the higher prevalence of positive cases in backyards and LBMs requires that all stakeholders be aware of the up-to-date biosafety and biosecurity recommendations. Continuous monitoring and early detection of new cases will help to reduce the risk of virus spread. Finally, updated genetic features using whole genomic sequencing of avian influenza viruses circulating in Egypt is essential to better understand virus evolution and the mechanisms of spread to neighboring countries.

## Figures and Tables

**Table 1:** Key amino acid analysis of the Egyptian HPAI H5N8 viruses among different proteins.

	HA ^#^							NA Stalk Del	PB2		PB1-F2 Length	M “Amantadine Resistance Markers”					NS1 Length
	Receptor Binding Sites						Cleavage Site		627	701							
	103	129	186	221	222	224						26	27	30	31	34	
Duck/Jiangsu/k1203/2010	H	L	E	G	Q	G	PLREKRRKRGLF	No	E	D	90	L	V	A	N	G	225
A/duck/Zhejiang/6D18/2013	H	L	E	G	Q	G	PLREKRRKRGLF	No	E	D	52	L	V	A	N	G	230
A/turkey/Germany/AR3390-L00939/2014	H	L	E	G	Q	G	PLRERRRKRGLF	No	E	D	52	L	V	A	N	G	237
A/great_crested_grebe/Uvs-Nuur_Lake/341/2016	H	L	E	G	Q	G	PLREKRRKRGLF	No	E	D	52	L	V	A	S	G	230
A/chicken/Iraq/1/2020	H	L	E	G	Q	G	PLREKRRKRGLF	No	E	D	52	L	V	A	S	G	217
A/barnacle_goose/Netherlands/20017557-002/2020	H	L	E	G	Q	G	PLREKRRKRGLF	No	E	D	52	L	V	A	S	G	217
A/domestic_duck/Germany-NI/AI03099/2021	H	L	E	G	Q	G	PLKEKRRKRGLF	No	E	D	52	L	V	A	S	G	217
A/common-coot/Egypt/CA285/20165	H	L	E	G	Q	G	PLREKRRKRGLF	No	E	D	52	L	V	A	S	G	217
A/chicken/Egypt/AF14/2020 *	H	L	E	G	Q	G	PLREKRRKRGLF	No	E	D	52	L	V	A	S	G	230
A/chicken/Egypt/AF12/2020 *	H	L	E	G	Q	G	PLREKRRKRGLF	No	E	D	52	L	V	A	S	G	230
A/chicken/Egypt/FAOS18E/2020 *	H	**F**	E	G	Q	G	PLREKRRKRGLF	No	E	D	52	L	V	A	S	G	230
A/turkey/Egypt/FAOSG6/2020 *	H	L	E	G	Q	G	PLREKRRKRGLF	No	E	D	52	L	V	A	S	G	230

^#^ H5 numbering. * Viruses for which whole genome sequences were obtained in this study.

**Table 2:** Amino acid sites under positive selection pressure estimated using different analytical methods in both HA (H5 numbering) and NA. Amino acids (a.a) detect in both methods are shown in bold.

Gene	a.a Residue	MEME		FEL	
		(*p*-Value ≤ 0.05)	Number of Branches under Episodic Selection	(*p*-Value ≤ 0.05)	LRT
HA gene	**23**	**0.05**	**1**	**0.04**	**4.5**
	**88**	**0.04**	**2**	**0.03**	**4.9**
	**94**	**0.04**	**9**	**0.03**	**4.8**
	175	0.01	7		
	195	0.02	4		
	438	0	1		
NA gene	**8**	**0.02**	**8**	**0.011**	**6.471**
	19	0.04	1		
	**29**	**0.05**	**6**	**0.0347**	**4.462**
	**37**	**0.04**	**6**	**0.0204**	**5.374**
	**38**	**0.02**	**5**	**0.0118**	**6.335**
	65	0.03	0		
	66			0.0358	4.408
	71			0.0323	4.584
	79	0.02	2		
	**80**	**0.01**	**7**	**0.0101**	**6.62**
	**89**			**0.0429**	**4.098**
	**151**	**0.03**	**5**	**0.0394**	**4.244**
	177			0.0232	5.153
	213			0.045	4.019
	**244**	**0.02**	**6**	**0.0191**	**5.492**
	**245**	**0.05**	**7**	**0.0402**	**4.211**
	**254**	**0.03**	**7**	**0.0339**	**4.501**
	**269**	**0.04**	**4**	**0.0453**	**4.008**
	270			0.0484	3.896
	**300**	**0.01**	**7**	**0.0026**	**9.046**
	311	0.02	1		
	**314**			**0.0444**	**4.041**
	350			0.0392	4.253
	**382**	**0.02**	**7**	**0.0049**	**7.917**

## Data Availability

The obtained sequences in this study were submitted to GenBank under the accession number shown in Supplementary Table S1.

## Supplementary Materials

The following supporting information can be downloaded at: https://www.mdpi.com/article/10.3390/v14081758/s1. Table S1: Number of examined commercial farm cases 2019–2021 for H5 avian influenza viruses. Table S2: Epidemiological data and accession number of sequenced H5 viruses in Egypt.

## References

[1] Nuñez I.A., Ross T.M. (2019). A review of H5Nx avian influenza viruses. Ther. Adv. Vaccines Immunother..

[2] Pohlmann A., King J., Fusaro A., Zecchin B., Banyard A.C., Brown I.H., Byrne A.M.P., Beerens N., Liang Y., Heutink R. (2022). Has Epizootic Become Enzootic? Evidence for a Fundamental Change in the Infection Dynamics of Highly Pathogenic Avian Influenza in Europe, 2021. mBio.

[3] Zhao K., Gu M., Zhong L., Duan Z., Zhang Y., Zhu Y., Zhao G., Zhao M., Chen Z., Hu S. (2013). Characterization of three H5N5 and one H5N8 highly pathogenic avian influenza viruses in China. Vet. Microbiol..

[4] Lee Y.-J., Kang H.-M., Lee E.-K., Song B.-M., Jeong J., Kwon Y.-K., Kim H.-R., Lee K.-J., Hong M.-S., Jang I. (2014). Novel Reassortant Influenza A(H5N8) Viruses, South Korea, 2014. Emerg. Infect. Dis..

[5] Bevins S.N., Dusek R.J., White C.L., Gidlewski T., Bodenstein B., Mansfield K.G., DeBruyn P., Kraege D., Rowan E., Gillin C. (2016). Widespread detection of highly pathogenic H5 influenza viruses in wild birds from the Pacific Flyway of the United States. Sci. Rep..

[6] Globig A., Staubach C., Sauter-Louis C., Dietze K., Homeier-Bachmann T., Probst C., Gethmann J., Depner K.R., Grund C., Harder T.C. (2017). Highly Pathogenic Avian Influenza H5N8 Clade 2.3.4.4b in Germany in 2016/2017. Front. Vet. Sci..

[7] Lewis N.S., Banyard A.C., Whittard E., Karibayev T., Al Kafagi T., Chvala I., Byrne A., Meruyert Akberovna S., King J., Harder T. (2021). Emergence and spread of novel H5N8, H5N5 and H5N1 clade 2.3.4.4 highly pathogenic avian influenza in 2020. Emerg. Microbes Infect..

[8] Lycett S.J., Pohlmann A., Staubach C., Caliendo V., Woolhouse M., Beer M., Kuiken T. (2020). Genesis and spread of multiple reassortants during the 2016/2017 H5 avian influenza epidemic in Eurasia. Proc. Natl. Acad. Sci. USA.

[9] Napp S., Majó N., Sánchez-Gónzalez R., Vergara-Alert J. (2018). Emergence and spread of highly pathogenic avian influenza A(H5N8) in Europe in 2016-2017. Transbound. Emerg. Dis..

[10] Fusaro A., Zecchin B., Vrancken B., Abolnik C., Ademun R., Alassane A., Arafa A., Awuni J.A., Couacy-Hymann E., Coulibaly M.B. (2019). Disentangling the role of Africa in the global spread of H5 highly pathogenic avian influenza. Nat. Commun..

[11] WHO (2021). Human Infection with Avian Influenza A (H5N8)—The Russian Federation.

[12] Selim A.A., Erfan A.M., Hagag N., Zanaty A., Samir A.H., Samy M., Abdelhalim A., Arafa A.A., Soliman M.A., Shaheen M. (2017). Highly Pathogenic Avian Influenza Virus (H5N8) Clade 2.3.4.4 Infection in Migratory Birds, Egypt. Emerg. Infect. Dis..

[13] Yehia N., Naguib M.M., Li R., Hagag N., El-Husseiny M., Mosaad Z., Nour A., Rabea N., Hasan W.M., Hassan M.K. (2018). Multiple introductions of reassorted highly pathogenic avian influenza viruses (H5N8) clade 2.3.4.4b causing outbreaks in wild birds and poultry in Egypt. Infect. Genet. Evol. J. Mol. Epidemiol. Evol. Genet. Infect. Dis..

[14] Hassan K.E., Saad N., Abozeid H.H., Shany S., El-Kady M.F., Arafa A., El-Sawah A.A.A., Pfaff F., Hafez H.M., Beer M. (2020). Genotyping and reassortment analysis of highly pathogenic avian influenza viruses H5N8 and H5N2 from Egypt reveals successive annual replacement of genotypes. Infect. Genet. Evol. J. Mol. Epidemiol. Evol. Genet. Infect. Dis..

[15] Yehia N., Hassan W.M.M., Sedeek A., Elhusseiny M.H. (2020). Genetic variability of avian influenza virus subtype H5N8 in Egypt in 2017 and 2018. Arch. Virol..

[16] Salaheldin A.H., El-Hamid H.S., Elbestawy A.R., Veits J., Hafez H.M., Mettenleiter T.C., Abdelwhab E.M. (2018). Multiple Introductions of Influenza A(H5N8) Virus into Poultry, Egypt, 2017. Emerg. Infect. Dis..

[17] Moatasim Y., Kandeil A., Aboulhoda B.E., El-Shesheny R., Alkhazindar M., AbdElSalam E.T., Kutkat O., Kamel M.N., El Taweel A.N., Mostafa A. (2019). Comparative Virological and Pathogenic Characteristics of Avian Influenza H5N8 Viruses Detected in Wild Birds and Domestic Poultry in Egypt during the Winter of 2016/2017. Viruses.

[18] Hagag N.M., Erfan A.M., El-Husseiny M., Shalaby A.G., Saif M.A., Tawakol M.M., Nour A.A., Selim A.A., Arafa A.S., Hassan M.K. (2019). Isolation of a Novel Reassortant Highly Pathogenic Avian Influenza (H5N2) Virus in Egypt. Viruses.

[19] Hassan K.E., King J., El-Kady M., Afifi M., Abozeid H.H., Pohlmann A., Beer M., Harder T. (2020). Novel Reassortant Highly Pathogenic Avian Influenza A(H5N2) Virus in Broiler Chickens, Egypt. Emerg. Infect. Dis..

[20] Beerens N., Heutink R., Harders F., Roose M., Pritz-Verschuren S.B.E., Germeraad E.A., Engelsma M. (2021). Incursion of Novel Highly Pathogenic Avian Influenza A(H5N8) Virus, the Netherlands, October 2020. Emerg. Infect. Dis..

[21] Amer F., Li R., Rabie N., El-Husseiny M.H., Yehia N., Hagag N.M., Samy M., Selim A., Hassan M.K., Hassan W.M.M. (2021). Temporal Dynamics of Influenza A(H5N1) Subtype before and after the Emergence of H5N8. Viruses.

[22] Kandeil A., Hicks J.T., Young S.G., El Taweel A.N., Kayed A.S., Moatasim Y., Kutkat O., Bagato O., McKenzie P.P., Cai Z. (2019). Active surveillance and genetic evolution of avian influenza viruses in Egypt, 2016-2018. Emerg. Microbes Infect..

[23] Hassan K.E., El-Kady M.F., El-Sawah A.A.A., Luttermann C., Parvin R., Shany S., Beer M., Harder T. (2019). Respiratory disease due to mixed viral infections in poultry flocks in Egypt between 2017 and 2018: Upsurge of highly pathogenic avian influenza virus subtype H5N8 since 2018. Transbound. Emerg. Dis..

[24] Spackman E., Senne D.A., Bulaga L.L., Myers T.J., Perdue M.L., Garber L.P., Lohman K., Daum L.T., Suarez D.L. (2003). Development of real-time RT-PCR for the detection of avian influenza virus. Avian Dis..

[25] Hoffmann B., Hoffmann D., Henritzi D., Beer M., Harder T.C. (2016). Riems influenza a typing array (RITA): An RT-qPCR-based low density array for subtyping avian and mammalian influenza a viruses. Sci. Rep..

[26] Singapore Tourism Board Chapter 2.3.4. Avian Influenza. http://www.oie.int/fileadmin/Home/eng/Health_standards/tahm/2.03.04_AI.pdf.

[27] Hoper D., Hoffmann B., Beer M. (2009). Simple, sensitive, and swift sequencing of complete H5N1 avian influenza virus genomes. J. Clin. Microbiol..

[28] Katoh K., Standley D.M. (2013). MAFFT multiple sequence alignment software version 7: Improvements in performance and usability. Mol. Biol. Evol..

[29] Nguyen L.T., Schmidt H.A., von Haeseler A., Minh B.Q. (2015). IQ-TREE: A fast and effective stochastic algorithm for estimating maximum likelihood phylogenies. Mol. Biol. Evol..

[30] Weaver S., Shank S.D., Spielman S.J., Li M., Muse S.V., Kosakovsky Pond S.L. (2018). Datamonkey 2.0: A Modern Web Application for Characterizing Selective and Other Evolutionary Processes. Mol. Biol. Evol..

[31] Cai Z., Ducatez M.F., Yang J., Zhang T., Long L.P., Boon A.C., Webby R.J., Wan X.F. (2012). Identifying antigenicity-associated sites in highly pathogenic H5N1 influenza virus hemagglutinin by using sparse learning. J. Mol. Biol..

[32] Mair C.M., Ludwig K., Herrmann A., Sieben C. (2014). Receptor binding and pH stability-How influenza A virus hemagglutinin affects host-specific virus infection. Biochim. Et Biophys. Acta (BBA)-Biomembr..

[33] Jeong S., Lee D.H., Kwon J.H., Kim Y.J., Lee S.H., Cho A.Y., Kim T.H., Park J.E., Lee S.I., Song C.S. (2020). Highly Pathogenic Avian Influenza Clade 2.3.4.4b Subtype H5N8 Virus Isolated from Mandarin Duck in South Korea, 2020. Viruses.

[34] Kandeil A., Moatasim Y., El Taweel A., El Sayes M., Rubrum A., Jeevan T., McKenzie P.P., Webby R.J., Ali M.A., Kayali G. (2022). Genetic and Antigenic Characteristics of Highly Pathogenic Avian Influenza A(H5N8) Viruses Circulating in Domestic Poultry in Egypt, 2017-2021. Microorganisms.

[35] Salaheldin A.H., Elbestawy A.R., Abdelkader A.M., Sultan H.A., Ibrahim A.A., Abd El-Hamid H.S., Abdelwhab E.M. (2022). Isolation of Genetically Diverse H5N8 Avian Influenza Viruses in Poultry in Egypt, 2019–2021. Viruses.

[36] Kandeil A., Kayed A., Moatasim Y., Webby R.J., McKenzie P.P., Kayali G., Ali M.A. (2017). Genetic characterization of highly pathogenic avian influenza A H5N8 viruses isolated from wild birds in Egypt. J. Gen. Virol.

[37] Chong Y., Ikematsu H. (2018). Is seasonal vaccination a contributing factor to the selection of influenza epidemic variants?. Hum. Vaccin. Immunother..

[38] Duvvuri V.R., Duvvuri B., Cuff W.R., Wu G.E., Wu J. (2009). Role of positive selection pressure on the evolution of H5N1 hemagglutinin. Genom. Proteom. Bioinform..

[39] Li W., Shi W., Qiao H., Ho S.Y., Luo A., Zhang Y., Zhu C. (2011). Positive selection on hemagglutinin and neuraminidase genes of H1N1 influenza viruses. Virol. J..

[40] Zeng X.-Y., He X.-W., Meng F., Ma Q., Wang Y., Bao H.-M., Liu Y.-J., Deng G.-H., Shi J.-Z., Li Y.-B. (2022). Protective efficacy of an H5/H7 trivalent inactivated vaccine (H5-Re13, H5-Re14, and H7-Re4 strains) in chickens, ducks, and geese against newly detected H5N1, H5N6, H5N8, and H7N9 viruses. J. Integr. Agric..

