# Supplementary material for: Molecular Epidemiology and Evolutionary Analysis of Avian Influenza A(H5) Viruses Circulating in Egypt, 2019–2021
Source: Viruses. 2022 Aug 11;14(8):1758. doi: 10.3390/v14081758 (PMC9415572; doi:10.3390/v14081758)
Supplement: Supplementary file 1 [file viruses-14-01758-s001.zip › viruses-1852866-supplementary.pdf]

Supplementary Information for:

Molecular epidemiology and evolutionary analysis of avian influenza A(H5) viruses circulating in Egypt, 2019-2021

Naglaa M. Hagag[1], Nahed Yehia[1], Mohamed H. El-Husseiny[1], Amany Adel[1], Azhar Gaber[1], Neveen Rabie[1], Mohamed Samy[1], Motaz Mohamed[1], Amal S. A. El Oksh[1], Abdullah Selim[1], Abdel-Sattar Arafa[1], Samah Eid[1], Momtaz A. Shahein[2], Mahmoud M. Naguib[1, 3,#]

[1]Reference Laboratory for Veterinary Quality Control on Poultry Production, Animal Health Research Institute, Agriculture Research Center, 12618 Giza, Egypt

[2]Virology department, Animal Health Research Institute, Agriculture Research Center, 12618 Giza, Egypt

[3]Zoonosis Science Center, Department of Medical Biochemistry and Microbiology, Uppsala University, 75121 Uppsala, Sweden

Supplementary Table S1. Number of examined commercial farm cases 2019-2021 for H5 avian influenza viruses.

Serial	Governorates	No. of swab samples	No. of tested cases	Positive cases
1	Alexandria	9111	298	0
2	Assiut	25627	1083	1
3	Aswan	1585	86	1
4	Beheira	85965	2150	0
5	Beni Suef	1905	96	5
6	Cairo	999	34	1
7	Dakahlia	57532	5397	4
8	Domietta	3201	203	0
9	Fayoum	9477	403	0
10	Gharbia	30184	1950	3
11	Giza	42525	1353	4
12	Ismailia	13284	593	0
13	Kafer el-Sheikh	7970	627	0
14	Luxor	1383	101	0
15	Matrouh	4122	106	0
16	Menia	14225	1134	4
17	Menofia	45664	4228	2
18	North Sinai	581	28	0
19	Port Said	1608	73	0
20	Qalyubia	59967	5673	9
21	Qena	2920	159	0
22	Red sea	6699	252	0
23	Sharqia	48110	2394	1
24	Sohag	5607	271	1
25	South Sinai	436	19	0
26	Suez	3394	116	0
27	unkown	403	39	0
28	Wady Gaded	21613	1293	1
Total		506097	30159	37

Supplementary Table S2. Epidemiological data and accession number of sequenced viruses.

Serial	Sample ID	Date	Governorate	Source	Surveillance	Accession no*
1	A/chicken/Egypt/FAO-SGI_10/7/2019	11-01-2019	Dakahlia	farm	active	ON024710
2	A/duck/Egypt/SMG6/2/2019	07-02-2019	Port Said	backyard	active	ON024714
3	A/chicken/Egypt/AL2/2/2019	13-02-2019	Aswan	backyard	passive	ON024696, ON015038
4	A/duck/Egypt/FAO-SGI_11/7/2019	15-02-2019	Dakahlia	market	active	ON024711
5	A/duck/Egypt/SMG7/2/2019	24-02-2019	Dakahlia	farm	active	ON024715
6	A/duck/Egypt/SD36/3/2019	13-03-2019	Beheira	backyard	active	ON024713
7	A/turkey/Egypt/FAO-SGI_22/7/2019	14-03-2019	Dakahlia	farm	active	ON024712
8	A/chicken/Egypt/V1345/1/2019	21-03-2019	Wadi Gaded	farm	active	ON024716, ON015023
9	A/duck/Egypt/FAO-S32/5/2019	08-05-2019	Qalyubia	market	active	ON024704, ON015040
10	A/duck/Egypt/FAO-S33/5/2019	08-05-2019	Qalyubia	market	active	ON024705, ON015036
11	A/turkey/Egypt/FAO-S10/5/2019	20-05-2019	Giza	market	active	ON024700, ON015035
12	A/duck/Egypt/FAO-S6/6/2019	05-06-2019	Giza	market	active	ON024699, ON015039
13	A/duck/Egypt/FAO-S17/6/2019	17-06-2019	Giza	market	active	ON024701
14	A/turkey/Egypt/FAO-S18/6/2019	17-06-2019	Qalyubia	market	active	ON024702, ON015034
15	A/duck/Egypt/FAO-S24/8/2019	07-08-2019	Qalyubia	market	active	ON024703, ON015031
16	A/duck/Egypt/FAO-S37/8/2019	26-08-2019	Qalyubia	market	active	ON024706, ON015030
17	A/turkey/Egypt/FAO-S42/9/2019	09-09-2019	Qalyubia	market	active	ON024707
18	A/chicken/Egypt/FAO-SG21/9/2019	26-09-2019	Dakahlia	market	active	ON024709
19	A/chicken/Egypt/AL6/10/2019	06-10-2019	Sohag	farm	passive	ON024697, ON015037
20	A/chicken/Egypt/AL7/10/2019	26-10-2019	Aswan	backyard	passive	ON024695
21	A/duck/Egypt/FAO-S76/11/2019	26-11-2019	Qalyubia	market	active	ON024708
22	A/chicken/Egypt/F565/12/2019	05-12-2019	Cairo	farm	passive	ON024698
23	A/chicken/Egypt/AI9/12/2019	30-12-2019	Ismailia	backyard	passive	ON024682
24	A/duck/Egypt/FAOS1/1/2020	05-01-2020	Qalyubia	market	active	ON024683
25	A/chicken/Egypt/VG37/1/2020	09-01-2020	Dakahlia	farm	active	ON024694
26	A/duck/Egypt/FAOSG3/1/2020	13-01-2020	Domietta	market	active	ON024689
27	A/turkey/Egypt/FAOS5/1/2020	14-01-2020	Al Giza	market	active	ON024688
28	A/turkey/Egypt/FAOSG5/1/2020	19-01-2020	Dakahlia	market	active	ON024690, ON015029
29	A/turkey/Egypt/FAOSG6/2020	26-01-2020	Domietta	market	active	ON024691,ON015044, ON015108, ON015758, ON016015, ON017779, ON024408, ON024645
30	A/chicken/Egypt/FAOSG11/2/2020	13-02-2020	Dakahlia	market	active	ON024692, ON015028
31	A/duck/Egypt/AI2/2/2020	18-02-2020	Ismailia	backyard	passive	ON024681
32	A/chicken/Egypt/FAOS18E/2020	19-02-2020	Al Gharbia	market	active	ON024685, ON015043, ON015107, ON015757, ON016016, ON017778, ON024407, ON024644
33	A/duck/Egypt/FAOSG14/2/2020	24-02-2020	Domietta	market	active	ON024693, ON015027
34	A/chicken/Egypt/FAOS20/3/2020	01-03-2020	Qalyubia	market	active	ON024686, ON015032
35	A/chicken/Egypt/AF12/2020	09-04-2020	Beni Suef	farm	passive	ON024679, ON015041, ON015105, ON015755, ON016013, ON017776, ON024405, ON024642
36	A/duck/Egypt/FAOS13/2/2020	02-05-2020	Gharbia	market	active	ON024684, ON015026
37	A/chicken/Egypt/AF14/2020	04-05-2020	Beni Suef	farm	passive	ON024680, ON015042, ON015106, ON015756, ON016014, ON017777, ON024406, ON024643
38	A/turkey/Egypt/FAOS24/3/2020	13-05-2020	Qalyubia	market	active	ON024687
39	A/chicken/Egypt/4A/2021	22-01-2021	Giza	farm	active	ON024718
40	A/turkey/Egypt/3FAO/SL/2021	29-01-2021	Sohag	market	active	ON024723
41	A/duck/Egypt/91F/2021	03-02-2021	Qalyubia	farm	passive	ON024719
42	A/duck/Egypt/4FAO/SL/2021	23-02-2021	Luxor	farm	active	ON024724
43	A/chicken/Egypt/304F/2021	29-03-2021	Dakahlia	farm	active	ON024720
44	A/turkey/Egypt/18FAO/S/2021	14-04-2021	Giza	market	active	ON024721
45	A/duck/Egypt/11FAO/SI/2021	22-04-2021	Ismalia	market	active	ON024725
46	A/turkey/Egypt/2A/2021	22-04-2021	Menia	farm	passive	ON024717
47	A/chciken/Egypt/19FAO/S/2021	25-04-2021	Giza	market	active	ON024722

* GenBank Accession number
